# N-acetylcysteine counteracts increased brain excitatory/inhibitory balance following maternal high-fat diet and restores emotional and cognitive profiles in adult mouse offspring

**DOI:** 10.1192/j.eurpsy.2024.217

**Published:** 2024-08-27

**Authors:** C. Musillo, M. Samà, B. Collacchi, M. A. Ajmone-Cat, R. De Simone, K. C. Creutzberg, M. A. Riva, A. Berry, F. Cirulli

**Affiliations:** ^1^Center for behavioral sciences and mental health; ^2^Dept. Neuroscience, Istituto Superiore di Sanità, Rome; ^3^Dept. Pharmacological and Biomolecular Sciences, University of Milan, Milan, Italy

## Abstract

**Introduction:**

High-fat diet (HFD) consumption during pregnancy can shape fetal brain development, increasing susceptibility to mental disorders. Nevertheless, the mechanisms underlying these negative outcomes remain unclear.

**Objectives:**

We hypothesize that mHFD induces inflammation and oxidative stress (OS) in the fetal brain, disrupting excitatory/inhibitory (E/I) balance in the adult brain. This results in altered hypothalamic-pituitary-adrenal (HPA) axis reactivity, emotional regulation, and cognitive function. We tested the ability of N-acetyl-cysteine (NAC) - a powerful anti-oxidant and anti-inflammatory compound - to counteract mHFD effects.

**Methods:**

Our mHFD model consists of female C57BL/6N mice fed either HFD (fat 58%, carbohydrate 25.5%, and protein 16.4%) or control diet (CD, fat 10.5%, carbohydrate 73.1% and protein 16.4%) before and during pregnancy (13 weeks). After 5 weeks on diets, half of them received NAC (1g/kg) for 8 weeks, until delivery.

Gene expression of *Il-1b*, *Cd68*, *Tmem119*, *iNOS*, and *Arg1* was measured in fetal brains. Cognitive function and emotional phenotype were assessed in adult male and female offspring through the Morris Water Maze (MWM) and the Emergence test, respectively. HPA axis functionality was assessed by measuring plasma corticosterone levels by ELISA following acute stress. Gene expression of vesicular glutamate transporter 1 (*Vglut1*) and vesicular GABA transporter (*Vgat*) were assessed as markers of E/I balance.

**Results:**

Exposure to mHFD induced inflammation and OS in the fetal brain of both sexes, by increasing *Il-1b* and *iNOS/Arg1.* Additionally, *Cd68* and *Tmem119* were specifically increased in females. In adulthood, mHFD reduced latency to emerge from the shelter in the Emergence test in both sexes. In females, mHFD impaired cognitive function, reducing time spent in the MWM target zone, and increased HPA reactivity in response to acute stress. Furthermore, mHFD decreased *Vgat* expression in both sexes, resulting in an imbalanced *Vglut1/Vgat* ratio towards excessive excitatory input. Maternal NAC supplementation rescued this imbalance.

**Conclusions:**

Overall, these data show that mHFD increases inflammation and OS in fetal brains, with greater effects in female offspring, inducing alterations in the E/I neuronal balance with concomitant disruptions of the neuroendocrine system and the emotional and cognitive profiles during adulthood. The supplementation with NAC was effective in rescuing the E/I imbalance as well as the behavioral phenotype.

**Disclosure of Interest:**

None Declared

